# Trust-Based Model for the Assessment of the Uncertainty of Measurements in Hybrid IoT Networks

**DOI:** 10.3390/s20236956

**Published:** 2020-12-05

**Authors:** Piotr Cofta, Cezary Orłowski, Jacek Lebiedź

**Affiliations:** 1 Faculty of Telecommunications, Computer Science and Technology, UTP University of Science and Technology, 85-796 Bydgoszcz, Poland; 2 Institute of Management and Finance, WSB University in Gdansk, 80-266 Gdansk, Poland; corlowski@wsb.gda.pl; 3 Faculty of ETI, Gdansk University of Technology, 80-233 Gdansk, Poland; jacekl@eti.pg.edu.pl

**Keywords:** IoT network, uncertainty, sensor network, reputation, trust management

## Abstract

The aim of this paper is to introduce a NUT model (NUT: network-uncertainty-trust) that aids the decrease of the uncertainty of measurements in autonomous hybrid Internet of Things sensor networks. The problem of uncertainty in such networks is a consequence of various operating conditions and varied quality of measurement nodes, making statistical approach less successful. This paper presents a model for decreasing the uncertainty through the use of socially inspired metaphors of reputation, trust, and confidence that are the untapped latent information. The model described in the paper shows how the individual reputation of each node can be assessed on the basis of opinions provided by other nodes of the hybrid measurement network, and that this method allows to assess the extent of uncertainty the node introduces to the network. This, in turn, allows nodes of low uncertainty to have a greater impact on the reconstruction of values. The verification of the model, as well as examples of its applicability to air quality measurements are presented as well. Simulations demonstrate that the use of the model can decrease the uncertainty by up to 55% while using the EWMA (exponentially weighted moving average) algorithm, as compared to the reference one.

## 1. Introduction

Uncertainty of measurement is a complex technical and social phenomenon and is of great importance to the usefulness of any measurement, instrument, or the node of the measurement network. Therefore, various methods are used to assess and decrease uncertainty, addressing one or more sources of uncertainty, such as intrinsic uncertainties of the measuring process and device, uncertainties related to the transmission and processing of measurement data, to the design and operation of the network, as well as to the understanding of the problem itself.

Uncertainty is often viewed, and standardized, from the perspective of statistics [1]. That is, if for the given node of the network the representative sample of the divergence between the true and the actual measurement can be determined, then its distribution can be estimated and the variance of such distribution can be taken as the assessment and prediction of uncertainty. This holds true for as long as comparable measurements on comparable equipment can be conducted through a well-executed process, against some reference data. It is less applicable if such statistical analysis is hard to achieve, such as heterogeneous measurement networks, with its mix of nodes of varying quality and provenance and the absence of calibration process.

### 1.1. Approach

The authors approach uncertainty from the information-theoretic perspective of Hirschman [2]. In this approach, uncertainty is related to Shannon’s entropy, i.e., to the insufficiency of information from and about the system. Thus, the system of which more is known, is characterized by the lower uncertainty and the increase in available information should lead to the decrease in both entropy and uncertainty at the system level. Consequently, the reduction of uncertainty is contingent on obtaining and exploiting latent information from the system.

One has to distinguish between latent information that already exist in the system, and available information that can be actively used to reduce the uncertainty of measurements delivered by such system. The former is the inherent property of the system and cannot be increased without its re-design, while the increase in the latter is contingent, inter alia, on exploiting various models, one of which is presented in this paper. This model particularly addresses situations where nodes have unknown or variable characteristics.

The NUT model (network-uncertainty-trust), presented in this paper, focuses on two aspects of uncertainty:

- Decreasing uncertainty in heterogeneous measurement networks, specifically where the outcome is collated from several measurement results, and
- Assessing uncertainty introduced by individual nodes in such networks, so that nodes introducing high uncertainty can be dealt with.

Those situations are increasingly common, as inexpensive nodes allow for dense networks to be built on the basis of a limited pool of reference nodes. The authors anticipate that the emergence of hybrid, semi-professionally operated networks should shift the interest in uncertainty from the statistical assessment of individual sensors, into the decrease of the uncertainty in the whole network, using alternative methods and alternative sources of information.

Such an alternative is afforded by the use of the concept of reputation of the node that is the foundation of the model presented here. This reputation is additional information that can be extracted from the system, and represents the generalized certainty associated with the measurements conducted by the particular node, yet assessed not against references but against its peers. This link between reputation and uncertainty has been introduced e.g., in [3]. The knowledge of such information, while decreasing uncertainty at the network level, may have a positive effect on decreasing measurement errors as well.

It is worth noting that the node may be of low reputation without being malicious or defective, as opposed to several works [4,5,6,7], which focus mainly on increasing safety by identifying and eliminating malicious nodes. The presented model is inspired by research on social networks in which trust is used to build trust management systems (see [8,9] for a general overview and [10] for an overview of trust management in IoT). For this reason, it does not assume that IoT sensors have “will” and can “trust,” but only that the concept of trust is used as a metaphor to express certain peculiarities of a method.

### 1.2. Elements of Technical Novelty

The model proposed in this paper contributes to the decrease of the uncertainty and consequently to the improvement to the quality of measurement in IoT sensor networks in the following ways.

- It formalizes reputation in autonomous networks of IoT sensors;
- It demonstrates that reputation can be used, with good results, in situations where no authority (i.e., reference sensor or external calibration) is available.
- It proposes a specific model and algorithm that can be used to decrease uncertainty in heterogeneous sensor networks.
- It determines its performance, against the reference model, and its sensitivity to key parameters of the network.
- It demonstrates, through simulation, key properties of the model, including improvements in performance, in comparison to the reference model.

### 1.3. Taxonomy of Concepts Related to Measurement Networks

The taxonomy of relevant concepts is presented in order to introduce the reader to the specificity of the networks discussed in this paper. This paper uses approach that is partially taken from social sciences, therefore the taxonomy below consists of three parts: it first introduces concepts related to IoT networks, then discusses uncertainty in general, and finally discusses concepts related to social networks.

- Internet of Things (IoT) is a concept by which clearly identifiable items (“things”) can either directly or indirectly collect, process, or exchange data.
- IoT node—a measuring device in IoT networks that automatically connects to the network and supplies data.
- Measuring (sensor) node—a type of IoT node whose measuring system or its elements are designed to perform the measurements independently or in combination with one or more additional devices.
- Measuring (sensor) network—the IoT network comprised of measuring nodes, maintained to conduct measurements of a given physical phenomenon.
- Reference measuring stations (nodes)—stations that comply with the standards provided by the European Committee for Standardization (CEN) or by the International Organization for Standardization (ISO).
- Hybrid networks—measuring networks that comprise measuring nodes of which some are reference nodes while others do not satisfy the requirements for being reference nodes.
- PM10—a mixture of airborne particles with a diameter of not more than 10 μm. An important element of assessing the overall air quality.
- Calibration process (calibration)—all activities establishing the relationship between the values of the quantity indicated by a measuring instrument and the appropriate values of physical phenomenon defined by the standard of the measurement unit.
- Quality of the node—the extent by which both the measurement of the node and the opinions issued by the node are free from errors.
- Error—any discrepancy between the actual and the perceived or measured quantity.

Subsequently, the concept of uncertainty is clarified. There are several definitions of uncertainty, but those relevant to the paper are as follows:

- Colloquially, uncertainty is used to express any form of doubt about the measurement result, regardless of its source.
- In metrology, uncertainty is a parameter defining the variability of measurement results, indicating the dispersion of the measured quantity value that should be determined for each measurement. The measure of uncertainty can often be provided via statistical parameters.
- When statistics is used, uncertainty of a random variable is defined by its standard deviation (whether for population or for the sample) or by the confidence interval of such a variable.
- In relation to entropy and information theory, uncertainty is defined as the sum of the temporal and spectral Shannon entropies, as a measure of the lack of information about the system. This is the definition used throughout this paper.

Another set of constructs such as trust, reputation, and confidence is taken from social sciences and applied to sensor networks.

- Trust is used in this paper as a metaphor, not in its literal sense. That is, the paper does not assume that nodes have volition, or opinions as such. The definition of trust adopted here defines it as a metric expressing the similarity between the expected and actual behavior of B, according to A, in a form of a subjective probability [11].
- Reputation, in turn, is what is generally said or believed about the character or position of a person or thing [12]. The relationship between trust and reputation is not straightforward [13]. For the purposes of this paper, the reputation of a node is a common expectation, globally and actively maintained by the network, about the uncertainty it brings into the network: the higher the reputation, the lower the expected uncertainty of the node.
- Confidence is an extent of trust that a node has in its own ability to judge another particular node. The separation of trust and confidence is conceptually similar to the notion of “uncertain probabilities” [3], but without introducing the associated mathematical apparatus.

A comprehensive discussion of the various computational confidence expressions, including scopes used, operations, and ways to combine trust and uncertainty (trust) is provided in [14].

### 1.4. Structure

This paper starts with a description of the problem and the approach taken, followed by the description of the state-of-the-art in the field of uncertainty assessment in IoT networks. It is followed by the description of the model, an extended example, and the performance analysis of the model through simulation. The paper closes with a discussion and conclusions.

## 2. Problem Statement

Let us consider a hybrid Internet of Things network that measures a certain physical phenomenon. By design, it may consist of some high quality nodes (being characterized, among others, by low level of uncertainty) as well as of a number of high or unknown uncertainty ones. High quality nodes may provide reference measurements, but they may be also expensive and hard to maintain. The remaining nodes, on the other hand, may come cheap and aplenty, but their maintenance or re-calibration (even against reference nodes) may leave a lot to be desired, often being left to untrained staff or volunteers. Further, those nodes may employ various constructions and various measurement methods, making them unique and incomparable, thus invalidating statistical approach to determine the errors they may introduce.

**Figure 1:**
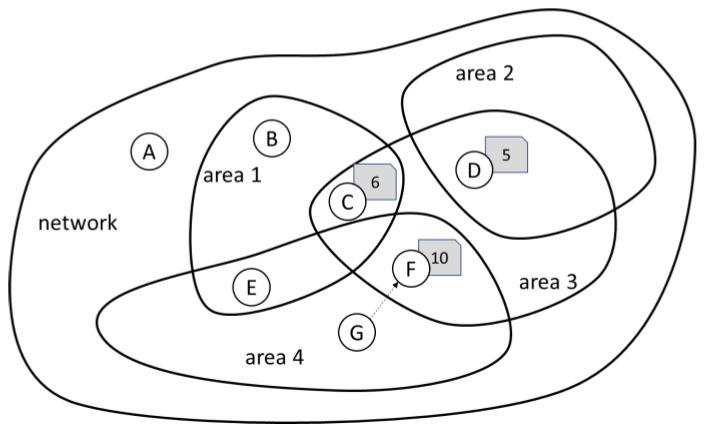
The network and its areas of interest.

The abundance of low-quality nodes makes the network dense in relation to the phenomenon it measures, so that users of the network may no longer be interested in measurements conducted by individual sensors, but rather in the value of the phenomenon across certain area of interest that may contain several nodes. For example, they may be interested in the electricity usage on a given street or air pollution in a given district. Figure 1 illustrates this concept: the area covered by the network can be divided into (possibly overlapping) areas of interest and some nodes may operate in more than one area. Nodes C, D, and F are shown together with the results of their most current measurements.

The network thus must consolidate measurements from individual nodes into the result relevant to the area. For example, in order to obtain information about area 3, the measurement from nodes C, D, and F must be consolidated. Without any other information available, all nodes provide their measurements as certain, even though the uncertainty of those measurements is likely to vary. Note that the information about calibration may not be available for those nodes, or may be outdated.

In the absence of any additional information, the consolidation of such measurements can be achieved by averaging the results, disregarding the unknown uncertainty of nodes. In this example, it will provide the outcome of seven (i.e., (6 + 5 + 10)/3). This strategy can be considered optimal, but the uncertainty (and consequently the error) associated with such calculation may depend on the particular mix of nodes.

It would be therefore beneficial to distinguish between nodes of low and high uncertainty, so that the outcome of those of low uncertainty will weight more on the outcome, preferably proportionally to the certainty they bring. That is, the process can be improved by replacing averaging with the linear combination of results (i.e., weighted average), with beneficial impact on both decreasing uncertainty and reducing errors. Further, as nodes contribute also to other areas, it would be possible to infer from their past behavior across the network whether they provide results with high or low uncertainty. For example, if C is usually correct, D is of somehow unknown quality while F tends to be wrong, the weighted outcome may be different, e.g., 6 × 0.7 + 5 × 0.2 + 10 × 0.1 which totals to 5.9 (relative weights were normalized).

Those weights that are attributed to individual nodes constitute information that has not been previously exploited, that is information that has a potential to decrease the uncertainty of the network. This paper proposes that the approximation of such weights, called “reputation” is in fact available from the system, and that it can be obtained through the process inspired by computational trust [15], specifically by allowing nodes to express their opinions (i.e., “trust”) about other nodes. That is, the network, as a whole, is in a position to determine, from individual observations, the relative reputation of its components that will form weights used later to calculate the weighted average over any area (or even used to judge the uncertainty associated with the individual node). The introduction of reputation in itself should decrease the Hirschman uncertainty.

This reasoning can be extended to situations where nodes do not measure exactly the same level of the phenomenon (i.e., they are not from the same area), but where they can infer from their own measurements what is the expected measurement of another node. For example, while node G is not expected to arrive to the measurement identical with the node F, its value can be somehow inferred by F, so that node F could be in the position to express its trust in G, once G’s measurements are known to F.

In the same way, reputation of individual nodes may be of use, as it provides an assessment of uncertainty that the network has to deal with, specifically if there is no statistical information about the node. That is, instead of dealing with the measurement of unknown provenance and quality, the network can exploit the shared belief of other nodes in the uncertainty of a given one.

This approach may also inform the design of the network to determine the appropriate ratio and relative location of nodes of various levels of uncertainty as well as the schedule of their deployment. Further, it may aid in the detection of nodes of particularly low reputation (i.e., high uncertainty) that should undergo the maintenance, specifically if those nodes change their quality in time.

The main objectives of introducing the NUT model are:

- Ensuring that data from nodes of high uncertainty do not significantly affect the uncertainty of results provided by the network as a whole.
- Identifying nodes that generate uncertain data, assuming they may be low-quality, defective, or may require maintenance.
- Providing design and maintenance guidelines regarding the appropriate mix of nodes of low and high uncertainty.

As an added benefit, such a network should be able to dynamically and autonomically respond to changes in the quality of individual nodes. Thus, the node of a gradually increasing uncertainty can be gradually discounted by other nodes by decreasing their reputation, only to be accepted once the maintenance or the repair will make its uncertainty decrease.

## 3. State-of-the-Art—The Problem of Measurement Uncertainty in IoT Networks

The research presented in this paper touches upon several concepts, so that the purpose of this section is mostly to review selected publications from various areas that collectively should provide an insight into the relevant state-of-the-art, and to indicate links between presented ideas and the proposed model. Those areas address (1) uncertainty of measurements, (2) uncertainty in sensor networks and information fields, (3) uncertainty and entropy from the perspective of information theory, (4) the use of reputation on IoT networks, (5) uncertainty of reputation, and (6) uncertainty as reputation, specifically in the IoT networks. In view of the number of articles in this area, this review is very selective. The focus is only on this current development of IoT network-based reputation systems, in particular the network sensor. From among the papers already known to the authors, a selection was done, complemented by keyword searches in databases such as Scopus, IEEE Browse, and others.

Uncertainty of a measurement can be defined as a doubt that exists about the outcome of the measurement (while error is a difference between the actual value and such outcome) [16]. It is sometimes compared to the trustworthiness or the quality of the measuring instrument, and this interpretation is close to this paper. The extent of uncertainty is attributable to particular sensors and is the result of the uncertainty of electrical or chemical processes that takes place within the sensor, as well as the instability of its environment or procedures [17]. There are several ways of assessing uncertainty that are based on using reference sources, environments, and protocols [18] to estimate the probability distribution function of measurements. The standard deviation is then taken as a measure of uncertainty. However, when statistical methods are not applicable, alternatives exist e.g., in a form of subjective probability [11], used in this paper.

The use of sensor networks, rather than individual sensors, introduces two additional aspects of uncertainty: (a) Uncertainty of data processing if data are fused from several sensors; and (b) uncertainty of data interpolation between measured points. That is, the outcome of having measurement results, already attributed with uncertainty, transferred, stored, and processed, may produce uncertainty higher or lower than the one attributable to sensors alone.

The conceptualization of uncertainty in the IoT networks is thoroughly discussed by [19], indicating its multi-faceted nature in complex, dynamic systems such as IoT networks for smart cities, listing not only technical but also social elements that affect the uncertainty. Ref. [20] identified several sources of uncertainty that are inherent in sensor networks and suggested the use of trust and reputation to estimate such uncertainty, as at least some of them may not be suitable for the statistical treatment.

For as long as the uncertainty of sensors is known, statistical methods can be applied to estimate the uncertainty of collated data. It is interesting to see that alternative solutions [21] such as fuzzy logic can be used as well. Another alternative can be provided by Bayesian inference, used, for e.g., in information fields to interpolate values of the fields between points where the actual measurement is conducted [22]. While here uncertainty is still related to statistics, it also refers to the insufficiency of information.

Information theory provides a complementing view on uncertainty, treating it as a lack of information [2]. Thus, the system of which everything is known should exhibit no uncertainty. Such internal uncertainty of the system can be assessed in the same way as its entropy, so that systems with higher entropy exhibit also higher uncertainty. Note, that not all of this information may be available to the external observer, thus making the observer perceive higher uncertainty than an internal one that there is. Further, [23] distinguishes between risk and uncertainty, arguing that situations of uncertainty apply only to situations where the probability distribution cannot be known. The approach taken in this paper uses the differentiation between information within the system and information known about the system, to use a model to extract additional information.

The use of reputation and trust has its sources in social networks [15]. However, once it is accepted that one node of the IoT network is capable of providing certain opinion about other nodes, the same concept, as well as the associated algorithms, can be applied also to IoT networks. The focus of the existing literature is more on identifying faulty or malicious nodes than on decreasing uncertainty. For that purpose, the population of nodes is often divided into “watchers” and the “observed” and only the views of the watchers count. There are several works that analyze and categorize various proposed trust models (i.e., reputation scoring algorithms) as applied to IoT networks. For example, [6] lists 13 algorithms, [10] describes 26 of them, while [24] no less than 80 algorithms, strongly postulating that only data from trustworthy nodes should be processed.

The special case is the trust-based routing (TBR) [25], as this approach resembles the one discussed in this paper. TBR addresses the selection of the best routing path in ad-hoc networks such as mobile ad-hoc networks or mesh networks. In TBR, nodes report their experience with their neighbors (i.e., whether they correctly forwarded the packet). They select one of the neighbors on the basis of reputation built collectively from those reports. Thus, the overall performance of the network is contingent on correctly attributing reputation to nodes, and usually outperforms random selection of routes [26]. TBR is used here to construct the reference (conventional) method to measure expected performance.

The fusion of opinions in reputation-based systems brings again the question of the uncertainty of opinions, and the resulting uncertainty of the reputation of nodes. Certain frameworks have been established to express, process, and reason about such uncertainty. For example [3] proposes the combination of Bayesian assessment of reputation combined with the calculation of uncertainty; [27] uses intervals and the appropriate algebra to the same effect, while [4,7] employ fuzzy logic.

Finally, modern research in social trust [28] regards trust as a way of dealing with uncertainty, specifically where information is insufficient or cannot be timely processed [13]. From this perspective, reputation can be considered a generalized certainty, derived from subjective probability [11] estimated out of their prior experiences. While not without its deficiencies, this approach is applicable in situations where the estimation of individual uncertainty of sensors and measurements cannot be established.

This brief analysis allows for the following conclusions:

- There is an established approach to uncertainty, based on statistics; but there are also alternative views and methods to deal with uncertainty, specifically in situations where the former is not applicable.
- The problem of uncertainty in IoT sensor networks can be dealt with using opinions, trust, and reputation.
- Sparse sensor networks are primarily concerned with the uncertainty of interpolation; dense networks are concerned with the uncertainty of data fusion.

## 4. The Network-Uncertainty-Trust (NUT) Model and Method

The objective of the NUT model is to minimize the overall uncertainty of the measurement at the network level by decreasing the impact that nodes with higher uncertainty may have on it. It makes use of the concepts of trust, uncertainty, and reputation, and applies them to the network of measuring nodes. It is built upon the following set of principles:

- (Overprovisioning) it is very likely that at any moment more than one node will be able to determine the value of the same phenomenon;
- (Persistence) past uncertainty of a node is an indicator of its future one;
- (Autonomy) the network can determine the uncertainty of nodes with no external support;
- (Curtailment) nodes that exhibit higher uncertainty should have lower impact on measurements at the network level;
- (Consensus) the network achieves consensus about the value of the measurement.

Admittedly, there may be other models that, within the frame of the same set of principles can be applied to the problem, but they are outside of the scope of this paper. With several possible models, partially presented earlier in this paper, the choice of the particular model is dictated by the main purpose of this paper: to validate that there is a decrease in uncertainty that results from using the reputation. The authors observed, even in the absence of any common benchmark, that the difference between the improvements offered by various models is marginal to the improvement over the situation where any model is used at all. Thus, while there is a scope for improvements and experimentation with various models, this one is sufficient to validate this approach to sensor networks.

Further, the authors expect that the significant improvement may result from addressing the network planning and maintenance, which differentiates the IoT networks from social ones. Early indications of the validity of this observation are included in this paper.

### 4.1. Assumptions for the Model and Limitations of Its Use

The development of the NUT model is based on the following assumptions and limitations:

- The network is a measurement (sensor) IoT network.
- The network is a hybrid one: some nodes introduce higher uncertainty than others do.
- The nodes are not provisioned with information regarding the prior uncertainty of themselves or other nodes.
- The nodes may use different measurement methods.
- The nodes both measure the phenomenon and observe other sensors.
- Each node may, from time to time, be in a position to anticipate, on the basis of its own measurement, what the measurement made by another node should be.
- There are no additional nodes in these networks, which are used only to observe the operation of others.
- External calibration, maintenance, or assessment is not readily available.

Given these assumptions, the use of metaphors of trust and reputation can be justified, as statistical methods may not be sufficient. In this way, the trust in a node can be equated with an individual perception of the trustworthiness of the measurements it produces (hence with the certainty of such measurements), and its reputation with a common belief in such trustworthiness. A case of special attention is the opinion, known from social networks, and its counterparts in IoT networks, as nodes do not engage in pseudo-random social or commercial meetings.

Let us start by observing that all nodes measure the same physical phenomenon directly. While the local instantaneous value of such a phenomenon may vary, the phenomenon itself is governed by physical principles that can be communicated to nodes in advance. Consequently, a node may, often, but not always, relate its own measurement to a measurement supplied by another node, provided there is a causal relationship between them. Specifically, several nodes may be in a position to measure exactly the same (or very similar) value of a phenomenon.

For example, if there are two electricity meters measuring the energy consumption of two houses on the same street, these meters may expect the power and current to be different (due to differences in instantaneous energy use), but the voltage and frequency will be reasonably identical. Likewise, if there are two sensors measuring the air pollution, one is some distance downwind from the other, it can be reasonably expected that the first one will have some delay readings that somehow follow the readings from the other, possibly allowing both sides to submit opinions.

It can therefore be expected that an opinion will be generated at least in response to the following situations:

- The observed node has provided some readings (measurements) and the observing node is able to determine if these measurements are as expected.
- The observed node did not give any measurement even though it was supposed to do so, because of, for example, being a faulty one.
- The observing node is able to tell that the status of an observed node has changed significantly in a way that affects its opinion about that node.

The opinion that one node provides about another one has always the same simple form, which can be expressed as “at level x, I am convinced that I trust the other sensor to a degree of y.” Thus, each opinion has two components: trust in the other sensor and confidence in its own ability to determine the extent of that trust.

The degree of trust is measured as an extent of belief that the other node behaves as expected, taking into account what this node knows. For example, if a node has measured a certain concentration of PM10 (particles 10 micrometers in diameter or less) and the wind is blowing straight at another node, that node only trusts the other node if what it reports follows a simple PM10 dispersion model.

The notion of a high and low quality node can be now expanded. The high quality node does not only provide measurements with low error, but also is able to provide opinions that reliably describe the state of the other node. Contrasting, low quality node provides measurement with high error while its judgement of other nodes is problematic as well. Note that barring lapses in the logic programmed into the node, those two features are inter-related. As the node can only base its opinion on its own measurement, it is likely that the node with high error will come to the incorrect conclusion about the other node.

The reputation of a node depends on the opinions other nodes hold about it, but opinions of those that have higher reputation weigh more. There is no circular relationship between the reputation and the opinions only because opinions are provided in an opportunistic way, possibly at random, and because older opinions weigh less toward the reputation, through the process of their gradual decay.

As sensors have to provide feedback on other sensors, they should be able to model a physical phenomenon in a way that is suitable for the limited processing capability of the IoT node. In the case of air pollution, the global dispersion models are complex (see [29]), but local dispersion models are simpler [30]. They usually rely on wind and temperature observations, something that is within the reach of an IoT node or may be complemented by other nodes. Note that the oversupply of sensors means that the local model takes precedence over the global model.

### 4.2. Reference Method

One of the objectives of this paper is to test the benefits resulting from the use of the proposed model. This section describes the way the reference method has been developed, in the absence of directly comparable ones.

The proposed model is applicable to the situations where there is a large number of sensors that warrant some forms of averaging of their results. Usually sensor networks deal with the inverse problem: the number of sensors is small relative to the required quantification of the phenomenon, so that it is the interpolation that is of primary interest, not averaging. Sensor networks therefore exclude some nodes only when they are faulty, by applying some versions of trust management, as described earlier.

However, such situations are trivial from the perspective of uncertainty: a node is either accepted as operational and results are taken “as is,” or it is considered faulty and its readings are ignored altogether. There is no concept of weighting the contribution of various nodes, as there is no concept of partial correctness. For that reason, those methods, albeit superficially similar, were not selected as reference ones.

There is, however, a structural similarity between the approach presented here and trust-based routing (TBR), described in earlier sections of this paper. This similarity allowed for the creation of the reference solution and an associated metric in line with the one used in TBR. TBR allows the network (usually the ad-hoc mobile network) to replace random strategy of packet forwarding by the strategy driven by the reputation of forwarding nodes. Such reputation is built from opinions submitted by nodes on the basis of interactions they had with their neighbors. Opinions, in turn, express generalized certainty (i.e., subjective probability) that the given node will correctly perform a task packet forwarding, regardless of the reason not to forward.

The metric used by TBR is the fraction of packets lost (i.e., not forwarded), relative to the random strategy (which forms their reference solution), for the same graph consisting of “good” and “bad” nodes.

The reference solution used here applies the similar idea, introducing the following reference method. The network has to measure the phenomenon that is constant across all nodes, i.e., all nodes should return the same value that is equal to the unity. However, owing to different levels of uncertainty, those readings may differ.

If the network has no understanding on the uncertainty of different nodes, it can only average all the readings, and the result of such averaging is what the reference model can do. If, however, the network knows relative uncertainty, it can use weighted average. The improvements in reconstructing the actual state of the phenomenon resulting from the use of the model can be then taken as the improvement attributable to the model. This concept has been formalized later in this paper in a form of a “unit response,” i.e., the network assessment of the phenomenon whose actual value is exactly one.

Note that even if use cases of the TBR and of the NUT are not fully comparable, the improvements offered by the TBR can be used at least to set some expectations. The fractional gain in performance is used here to express such improvements, calculated as the percentage improvement in unit response over the one without the model. Using [31] as a reference, one could expect improvements in the range of 40%, for networks where up to 50% nodes are of low quality (untrustworthy). Further, it can be expected that the unit response will be primarily the function of the distribution of the nodes of various levels of quality, but it can be also affected by other parameters such as graph density.

This paper also presents additional results related to alternative intended use of the model. These are the analysis of misclassification (in a way similar to the detection of faulty nodes) and the analysis of the response to rapid changes in the behavior of nodes (also related to the potential use in detection faulty nodes). For those two, no specific benchmark has been stablished, and they are for information only.

### 4.3. Model Formalization

Measurement methods. There is a set of measurement methods applicable to the same physical phenomenon such as, for e.g., the concentration of PM10 in the air or the current voltage in the grid. This formalization reflects the fact that nodes may apply measurement methods that are not directly comparable, as it happens e.g., for the PM10 nodes. We define this set as $M=\left\{m_{1}\ldots m_{k}\right\}$.

We define a conversion function $f^{m_{i},m_{j}}:V\to V$ (where *V* is a set of measured values) that allows converting values $v_{i}\in V$ measured according to method $m_{i}$ into values $v_{j}\in V$ measured according to method m*_j_*. The function may not be defined for all pairs of methods, in which case the conversion is not possible. We define a set of such functions as $F_{c}$.

Sensors and events. There is a network of sensor nodes that measure the same physical phenomenon, each using one of the specified measurement methods. We define the sensor node as a tuple, so that the set of sensor nodes is defined as $S=\left\{s_{1},\ldots ,s_{n}\right\};\;s_{i}=\left(p_{i},m_{i}\right)$, where

- $p_{i}\;$is the parameter of the sensor node, such as its identifier, manufacturer, or location,
- $m_{i}\;$is the measurement method used by the sensor node.

Within this formalization, we assume that one sensor node measures only one physical phenomenon. This limitation simplifies formalization without losing generality of the proposal. For multi-function sensors, a single sensor of this kind can be represented by several specialized sensor nodes.

At certain moments in time, each sensor node generates events that report the measurements of the physical phenomenon. We define a set of events $E=\left\{e_{1},\ldots ,e_{n}\right\};\;e_{i}=\left(s_{i},t_{i},v_{i}\right)$, where

- $s_{i}\;$is the sensor node;
- $t_{i}\;$is the moment in time where the reading has been taken, to the best knowledge of the sensor;
- $v_{i}\;$is the value of readings (note that the value should only be interpreted in the context of the measurement method used by the sensor node).

Time and ordering. Regarding time and ordering of events, we assume that

- Sensors have time synchronized well enough for the purpose of this model. That is, potential differences in current time have only negligible effect.
- Events are always completely ordered, so that no two events are simultaneous. If events appear to be physically performed at the same time, the formalization requests an additional form of ordering.

Opinions. The proposed algorithm works on the basis of opinions generated by any sensor about the past performance of other sensors. Those opinions form a set $O=\left\{o_{1}\ldots o_{q}\right\};\;o_{i}=\left(sa_{i}\in S,ta_{i},sb_{i}\in S,tb_{i},u_{i},c_{i}\right),$ where

- $sa_{i}\;$is the sensor that provides this opinion;
- $ta_{i}\;$is the moment in time when $s_{a}\;$provided the opinion;
- $sb_{i}\;$is the sensor the opinion is about;
- $t_{bi}\;$is the moment in time that the opinion refers to;
- $u_{i}\;$is the level of trust that $sa_{i}\;$had in $sb_{i}$ at $tb_{i}$;
- $c_{i}\;$is the $sa_{i}$’s confidence in its judgement about $sb_{i}$; note that $sa_{i}$ may exhibit different levels of confidence about different nodes, and different level of confidence about the same node at different points in time. This level of confidence may depend, e.g., on their relative distance, freshness of available opinions etc.

For example, if the first opinion will come at 120 from node A, is about node B, and it states that A is confident at the level of 0.8 that at 110 B could have been trusted to the extent of 0.9, the opinion will be denoted as $o_{1}=\left(A,120,110,0.9,0.8\right)$.

Reputation. The way the reputation is calculated requires determining the subset of opinions that relates to a given sensor’s performance before or at the point in time. That is, for a reputation of $s_{b}\;$at time $t_{k}$ it is necessary to determine the set $O^{s_{b},t_{k}}=\left\{o_{1},\ldots ,o_{n}\right\};o_{a}=\left(s_{a},t_{a},s_{b},t_{b},u_{\mathrm{ab}},c_{\mathrm{ab}}\right)where\;t_{b}\leq t_{k}$. This set contains all opinions about $s_{b}$ before the time $t_{k}$. Note that it is the set of the $s_{b}$ that must lie before $t_{k}$; the opinion can be later than $t_{k}$, as other sensors may be in the position to provide such an opinion only sometime later.

It is also necessary to define the decay function that will assure that opinions that are “older” weigh less on the overall reputation, so that the sensor can eventually recover from occasional faults. The format of this function may differ, as it may take into account not only the passage of time, but also e.g., the number of options that have been provided or other criteria. For that reason, we define it here only by its signature $f_{d}:\left\{\left(o_{i},t_{k}\right)\right\}\to \left[0\ldots 1\right]$. It can be, for example, the exponential decay function so that $f_{d}=e^{-\lambda \left(t_{k}-t_{a}\right)}$. where λ is the decay constant. Note that it is the time of opinion that matters, as it is the value of the opinion that decays.

Now the reputation of $s_{b}\;$at time $t_{k}\;$can be expressed as the weighted sum of opinions from $O^{s_{b},t_{k}}$. The proposed formula is a variant of a trust-based weighting scheme, using the EWMA (exponentially weighted moving average) [15].

(1) $$w_{b}=\frac{\sum_{a=1}^{n}\left(u_{ab}\times conf_{ab}\right)}{\sum_{a=1}^{n}conf_{ab}}$$

where

$$conf_{ab}=c_{ab}\times w_{a}\times f_{d}\left(o_{ab},t_{k}\right)$$

Note that $w_{a}$ is the reputation of the sensor that provided the opinion calculated *for* the time the opinion was provided, but calculated *at* the time this calculation was made. Therefore, if the opinion was provided at time $t_{1}$ and the formula was calculated at some later time $t_{2}$, then the reputation of the sensors that provided the opinion is calculated for $t_{1}$. However, this reputation will be affected by all the opinions about the provider that have been recorded up to $t_{2}$.

### 4.4. Special Cases and Architectural Notes

The applicability of this proposal depends on certain structural properties of the network. In particular, it requires opinions to be issued relatively randomly. If the pattern of opinions is more regular, this can lead to undesirable distortions.

The special situation arises when there is a relatively isolated pair of nodes. Assuming that one is of high quality, and the other one is of a low one, the former may correctly speak negatively of the latter, but also the latter may have a negative opinion about the former. This can lead to a variant of the liar’s paradox in which there is not enough information to determine the reputation of any of them. The proposed solution largely discounts opinions from both of them, which may seem unfair, but may benefit the network as a whole.

Extending this example to a group of nodes, the outcome depends on both the cardinality of both sets, as well as on the frequency of opinions, as demonstrated later in this paper. It is one of the reasons why the NUT model is applicable only to networks with certain randomness of opinions.

Another interesting situation arises when there is only one node that correctly identified particular change in the measured phenomenon, while the remaining ones were not able to do so. Assuming that the node is considered to be the quality one, this new value will affect the current result reported by the network, but it will also make the node’s reputation low, thus discounting its reported values from any further contribution.

It is one of the reasons why the NUT model applies to networks where several nodes may record the same value of the phenomenon. If the majority of nodes record the same value, it will be reported correctly. This situation is not uncommon to other types of networks and measurements, e.g., to data cleansing where potential outliers are outright removed from the data set.

The implementation of the proposition may rely on the central processing, where all sensors report their measurements to the central database, and a specific piece of software (“sensor agent”) issues opinions on behalf of them. This is the implementation assumed by this paper, and it is the implementation of the actual network [32].

It is also feasible to have the distributed implementation, where sensors locally broadcast their measurements and opinions (e.g., using wireless mesh networks), while the actual algorithm is fully distributed (see e.g., [33,34]).

## 5. An Extended Example of an Application of the Model

For the purpose of illustration, let us consider an example of a sensor network, intentionally simplified for this purpose. The measured phenomenon is the concentration of the PM10 in the air. Sensors use the simplistic model of the relationship between readings taken at different places. For illustration purposes only, the operation of the scheme can be summarized as follows.

- Sensors are located along the “x” axis at some locations. They are not evenly spaced. They have unconstrained access to the surrounding air.
- Readings are provided by sensors at different moments. Those moments are not synchronized among sensors and may not be evenly spaced in time.
- Wind blows exactly along the “x” axis at constant speed. Wind carries PM10 with it. There is no dispersion of PM10 as it travels with the wind.
- If there is a need to estimate the values in-between readings, the sensor uses linear interpolation of two adjacent readings. Uncertainty of the outcome of this interpolation grows with the distance from actual readings.
- Sensors operate only on existing readings and existing measurements of the environment (such as e.g., wind speed and direction). That is, sensors cannot make predictions.
- Observing sensors respond to their readings as they arrive. When the reading arrives, the sensor can generate as many opinions as it considers appropriate, taking any readings that are considered appropriate.

The example discussed below uses only two sensors, aptly named “sensor A” and “sensor B.” Sensor B is located some distance downwind from sensor A so that the changing concentration of PM10 is first measured by sensor A, and then sometime later the same concentration is measured by sensor B.

**Figure 2:**
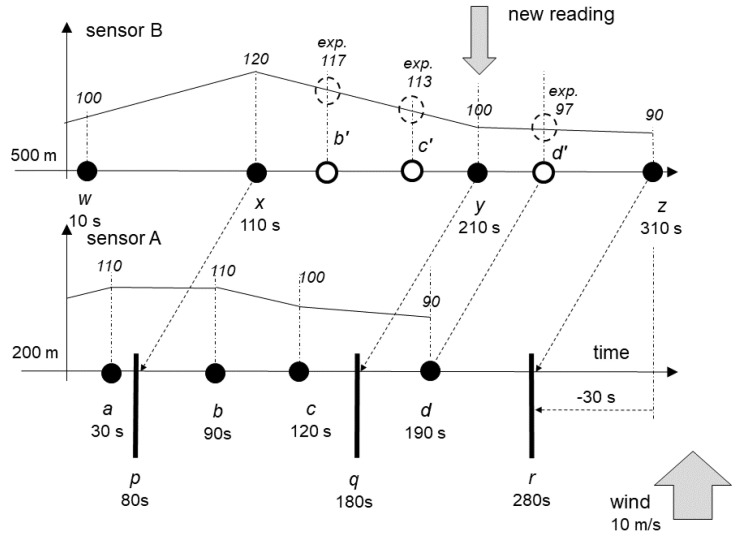
An example of the operation of the NUT (network-uncertainty-trust) model.

Both sensors are shown in Figure 2, together with some of their past readings. Horizontal axis represents the flow of time, with the new reading been made at 210 s from the start. Vertical axis represents the level of concentration of PM10 in the air at the location of the sensor, linearly interpolated between readings. Sensors are located at 200 m and at 500 m from a certain point, with wind blowing at 10 m/s exactly from A toward B, with no dispersion.

This example and the discussion below assume that opinions are generated by sensors’ agents, not by sensors themselves. Such agents have access to the database of readings of their own sensors, as well as to readings that arrived from other sensors.

Let us consider the situation depicted in Figure 1, where the new reading *y* is produced by sensor B at exactly 210 s. Note that the diagram contains also the reading *z*. Its role will be explained later, and for the moment, it should be ignored. The reading y is therefore the latest reading of sensor B. B already knows its past readings *w* and *x*, and readings *a*, *b*, *c*, and *d* from sensor A.

B now has to determine whether it can provide an opinion and what the opinion should be. The rule is that the opinion is always about the event that is in the past compared to the current one, accounting for any time offsets. As the wind blows at constant speed, whatever B decides about A, will refer to the past, i.e., it will be offset by 30 s to make it 180 s. This point is marked at the lower line that refers to A as *q*.

It is possible, yet unlikely, that there is a reading from A at 180 s, so that it exactly matches the *y* reading from B, accounting for time offset. It is more likely that there will be some readings from A close to this one. In order to determine the readings about which B can provide opinion, sensor B should determine the first of its readings that preceded *y*, in this case the reading *x*.

Now B can produce opinion about any reading of A that falls between *x* and *y*, considering time offset. As *y* (at 210 s) is mapped to the moment *q* (180 s) and *x* (at 110 s) to the moment *p* (80 s), B can determine that it can produce opinions about A’s readings *b* (90 s) and *c* (120 s).

In order to produce such opinions, B should interpolate the expected value from its own readings, resulting in *b’* (117) and *c’* (113), and then compare them with values at *b* (110) and *c* (100). The difference between the expected value and the one from the reading can be converted into the level of trust. If the difference is small, the opinion states that the sensor can be trusted. The exact function may be associated with the measurement method, as every method may have different sensitivity.

The level of confidence is related to the way the interpolation is done. With two readings far apart, the level of confidence is lower. The exact function that determines the level of confidence may depend on the sensor, as the sensor is aware of its normal reading pattern, i.e., the expected distance between the two adjacent readings.

It may be noticed that there may be an opportunity to generate one opinion more. When *y* arrives from B, A has both readings *c* and *d*, and *y* is mapped in-between those two. Consequently, A can provide opinion about B’s reading at *y*, by comparing its expected 97 with the actual value 90. While this requires A’s agent to watch new arrivals of readings from B, issuing this opinion is a valid opportunity.

As these readings are taken and processed centrally, not directly at sensors, it is possible, although hopefully unlikely, that readings from B will not arrive in order, because of, e.g., some delays in data transmission or processing. For example, it is possible that there is already a reading *z* that is later than *y* so that opinions were already provided about *b*, *c*, and d on the basis of *x* and *z*.

Once *y* arrives, there is an opportunity to generate new opinions, about readings b and c on the basis of the range *x* to *y*, and about the reading *d* on the basis of the range *y* to *z*. Consequently, readings *b*, *c*, and *d* will have two opinions each. Original opinions are likely to have lower level of confidence, as the distance between *x* and *z* is higher than between *x* and *y* or *y* and *z*. New opinions, of higher confidence, may have higher impact on the overall calculations.

## 6. Performance Analysis for Autonomous Heterogeneous Networks

While the proposed algorithm is a variant of the EWMA one, popularly used by reputation-based systems, its applicability to autonomous sensor networks requires additional analysis, as (to the knowledge of the authors) such applicability has never been demonstrated. Following its main objectives, the model should:

- Correctly determine the reputation of the sensor node, so that sensors of lower reputation have less impact on the outcome of the measurement,
- Help determine sensor nodes that are likely to be faulty, so that corrective actions can be taken,
- Eventually converge on the correct assessment of nodes, under various starting conditions.

Simulation was used, as the primary tool, for both verification of the method and the performance analysis. The random graph was used to simulate the sensor network, with its density being of the parameters. The graph was always populated by two different types of nodes: of low uncertainty (“trustworthy”) and of high uncertainty (“untrustworthy”). The choice of only two types of nodes allowed for a better understanding of basic relationships. The uncertainty itself was simulated by a fixed difference between the reported and the true value of the measurement. Alternative methods that are based on different distributions were also considered, but this one is characterized by its relative simplicity while delivering similar results.

In general, trustworthy nodes provided measurements with low uncertainty that were either exact, or not far off from the true value, untrustworthy ones provided results that were quite distant from the true one. One node determined its trust in another node by comparing the value it measured itself (that it took as a true one) and the observed value produced by another node. Nodes used exponential function $trustworthiness=e^{-\lambda \left|own-observed\right|}$ to convert the absolute difference between those two values into the assessment of trustworthiness. As a result, trustworthy nodes attributed trust to other trustworthy nodes and the lack of trust to untrustworthy ones, while untrustworthy nodes acted in the exactly opposite way.

The simulation concentrated on determining the characteristics of the algorithm that follows the expected use of the:

- Ability to decrease the impact of untrustworthy nodes on the overall measurement; quantified as the level of the unit response, i.e., the response of a whole network to the identical stimulus, for various fractions of trustworthy nodes;
- Ability to discern between trustworthy and untrustworthy nodes, specifically when untrustworthy nodes ceased to function properly, as a function of a fraction of trustworthy nodes;
- Ability to identify nodes that transited to the opposite role (specifically from being trustworthy to untrustworthy); quantified as a number of steps required to correct the classification of such nodes.

The simulation took into account the following properties of the network:

- Fraction *f* of trustworthy nodes in the network, quantified as a percentage of all nodes,
- Graph density, *g*, quantified as an average number of inbound edges (opinions) per node,
- Decay factor, quantified in the simulation by its inverse, the retention factor *r*.

### 6.1. The Simulation Algorithm

The implementation of the algorithm (Algorithm 1.) used for the simulation makes use of the fact that, assuming an equal duration of steps, the algorithm can be expressed in an iterative way. Apart from its trustworthiness at the current, ith step, $w_{j,f}^{i}$, the jth node retains all the past estimations of its trustworthiness, down to the initial one of 0.5. Further, r denotes the retention rate.

**Algorithm 1.** Simulation.

1. Generate n*f trustworthy and n*(1 − *f*) untrustworthy nodes

2. For each node *j* = *1* … *n* set $w_{j,f}^{0}$ to *0.5*

3. For each step, *i* = *1* … *m*

4.  For each node j=1…n simulate its measurement $v_{j,f}$ according to its parameters

5.  Remove old and generate new n*g edges to satisfy the required distribution
6.  For each edge from $s_{a}$ to
$s_{b}$ decorate the edge with:
7.   the opinion, calculated as $u_{ab}=e^{-\lambda \left|v_{a}-v_{b}\right|}$
8.   the fixed confidence $c_{ab}=1.0$

9.  For each node *j* = *1* … *n*

10.   Identify the set of inbound edges $e_{aj}\in E$

11.   Calculate $u_{j,f}^{i}=\underset{e_{aj}\in E}{\sum }\left(u_{aj}\times c_{aj}\times w_{a,f}^{i-1}\right)$ and
$d_{j,f}^{i}=\underset{e_{aj}\in E}{\sum }\left(c_{aj}\times w_{a,f}^{i-1}\right)$

12.   If $d_{j}^{i}\neq 0\;\mathrm{then}w_{j,f}^{i}=u_{j,f}^{i}/d_{j,f}^{i};$ otherwise
$w_{j,f}^{i}=0$

13.   Increment $w_{j,f}^{i}\;\mathrm{by}\;\overset{i}{\underset{k=0}{\sum }}w_{j,f}^{i-k}r^{k}$

14.  Report current values of
$w_{j,f}^{i}$ for all nodes, *j = 1 … n*


### 6.2. Simulation Results: Unit Response

Unit response is a primary metric to benchmark the algorithm. It is used here to reflect the certainty of the measurement at the network level, i.e., as a metric of the ability of the network to determine certain values out of current measurements, despite the fact that some nodes introduce more uncertainty than others do. It assumes the situation where all nodes of the network are supposed to measure the same value of exactly one. For the NUT model, the unit response is calculated as the weighted average of values from all nodes, where the weight is proportional to the reputation of the node. As a reference benchmark, the non-weighted average can be used, representing the behavior of the network without the model. Using the notation from the algorithm, the weighted average (*wa*) at the step *i*, for a given *f* (the fraction of trustworthy nodes) can be calculated as

(2) $$wa_{f}^{i}=\frac{\sum_{j=1}^{n}w_{j,f}^{i}\times v_{i,f}}{\sum_{j=1}^{n}w_{j,f}^{i}},\;$$

Note, that while the weighted average required simulations to establish values of $w_{j}^{i}$, the non-weighted average (*nwa*) for a given fraction of trustworthy nodes can be calculated analytically from the following formula, and does not depend on the simulation step:

(3) $$nwa_{f}=f\times v_{t}+\left(1-f\right)\times v_{u},\;$$

where

- f is a fraction of trustworthy nodes in the graph,
- $v_{t}$ is a value assumed always by the trustworthy (high quality) node,
- $v_{u}$ is a value assumed always by the untrustworthy (low quality) node.

For the model with only perfect nodes (high quality, with $v_{t}=1$), the response should be equal to one. Lower values indicate that the result is affected by some untrustworthy nodes that were not entirely screened off or that even high quality nodes were not able to attain the value of one. The gain from using the model can be observed as the increase of the unit response above the reference line that reflects the non-weighted average.

Fractional gain, used to determine the percentage improvement in performance, is calculated using the formula (4). The 20th step was used, as further improvements were negligible.

(4) $$gain_{f}=\left(wa_{f}^{20}-nwa_{f}\right)/nwa_{f},\;$$

#### 6.2.1. The Impact of Graph Density and Retention Factor on Unit Response

While the unit response is related mostly to the fraction of trustworthy nodes, it was necessary to determine the impact of remaining parameters: graph density and retention factor on unit response, to identify values that will be used throughout the simulation.

**Figure 3:**
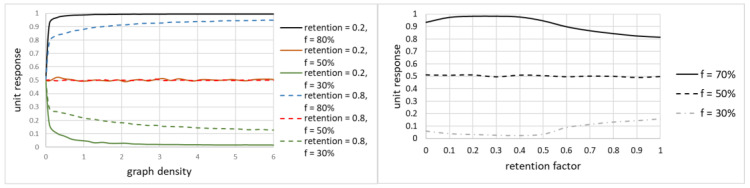
Unit response as a function of graph density (**left**) and retention factor (**right**).

Unit response is not sensitive to either one of those parameters. Figure 3 shows some of the simulations, for different fractions of trustworthy nodes. For graph density, its impact on unit response stabilizes above 3.0, the value that is achievable in networks. For the retention factor, values 0.1 to 0.4 maximized unit response for large fractions of trustworthy nodes, which was of an interest to the simulation. Therefore, for subsequent simulations, it was assumed that graph density is set to 3.0, while retention factor is set to 0.2.

#### 6.2.2. Unit Response as a Function of a Fraction of Trustworthy Nodes

The relationship between the fraction of trustworthy nodes and the unit response was the key element of simulation. This research assumed that by exploiting opinions and reputation it would be possible to achieve better results than by using simple averaging.

**Figure 4:**
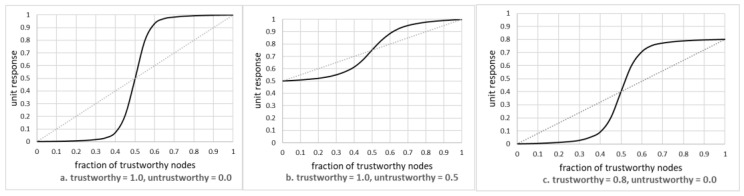
Unit response as a function of the fraction of trustworthy nodes.

This is shown in Figure 4 as three diagrams, reporting on the situation where the actual value of the phenomenon was equal to 1.0. All diagrams show the situation after 20 steps, as it was determined separately (see later in this paper) that subsequent improvements in performance were negligible.

The leftmost diagram shows the situation where the trustworthy node reported the correct value (i.e., $v_{t}=1.0$) while the untrustworthy one reported $v_{u}=0.0$, i.e., the situation that may emerge from untrustworthy nodes being faulty. The central one shows the mix of trustworthy nodes reporting the correct value, and some low quality nodes reporting 0.5 instead. Finally, the rightmost diagram shows the situation where there is a mix of medium quality trustworthy nodes (reporting 0.) and some faulty ones reported 0.0.

For each diagram, the dashed line represents the reference, i.e., the value that can be obtained by using the non-weighted average. Thus for any situation where the solid line is closer to 1.0 than the reference line, the model improved the results, while the inverse situation means that the model actually made the results worse.

#### 6.2.3. Fractional Gain

**Figure 5:**
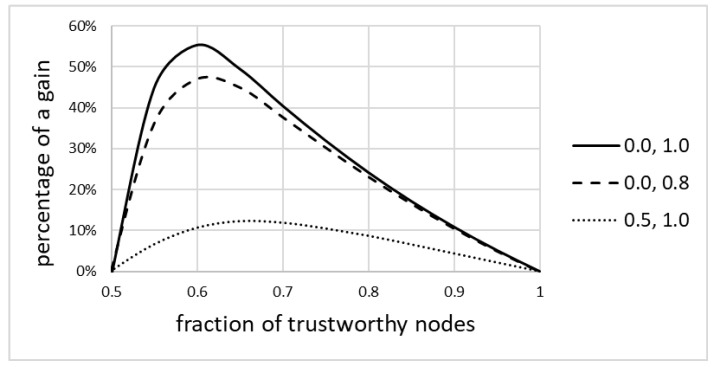
Fractional gain resulting from the use of the model.

Figure 5 shows the percentage (fractional) gain resulting from the use of the model, as a function of the fraction of trustworthy nodes, for various pairs of values $v_{u}$ and $v_{t}$ (see Equation (4)). Only the range of *f* = [*0.5*, *1.0*] is shown, as for lower values it was apparent that the model did not provide any advantage over the non-weighted average. The maximum gain reached about 55%, much better than that expected from the literature.

The following can be observed:

- The solid line always assumes a sigmoid-like shape. For fractions of trustworthy nodes below 50%, the model did not improve the readings, but it had a significant impact on improving those readings for larger fractions. Thus, the model provides benefits for networks with the relatively large fractions of trustworthy nodes. This finding is discussed in more detail later in this paper.
- Specifically, the model is good at discounting inputs from nodes that can be suspected of being faulty, or that may require maintenance. This finding is corroborated by the simulation of misclassification, presented later in this paper.
- The percentage gain over the non-weighted average can be as high as 55%, and it is the highest for the fraction of trustworthy nodes being close to 60%. The gain gradually disappears for larger fractions, as the absolute number of untrustworthy nodes decreases. Thus, benefits of the model are quite significant specifically for networks where the fraction of trustworthy nodes is not that high.
- The gain, both absolute and fractional, is more pronounced for networks where nodes significantly differ in quality. If untrustworthy nodes report values that are somehow correct (e.g., 0.5 instead of 1.0), then the improvement is still visible, but is of lesser value.
- The model cannot improve above the uncertainty of trustworthy nodes, i.e., it is not a substitute for having some quality nodes in the network. If, for example, the best node reports 0.8 instead of 1.0, then the model will report 0.8 in the best case. It can, however, as expected, reduce the impact of low quality nodes.

### 6.3. Simulation Results: Misclassification

Another intended use of the model is the ability to identify nodes that may require maintenance or replacement, i.e., the nodes that may be regarded as faulty. It was already demonstrated in the previous section that the input from those nodes is discounted. However, the detection of faulty nodes required additional simulations.

For the purpose of this simulation, the 0–1 classifier was introduced, with the threshold value set at 0.5. That is, nodes with trustworthiness greater than 0.5 were considered “good,” nodes with lower trustworthiness were treated as “bad.” The simulation focused on general misclassification, as both kinds of misclassification introduce the unwanted cost to the network. Taking a trustworthy node as a faulty one causes unnecessary servicing or calibration; allowing a faulty unit to go as a trustworthy one increases the uncertainty of the measurement, thus decreasing the usefulness of the network.

**Figure 6:**
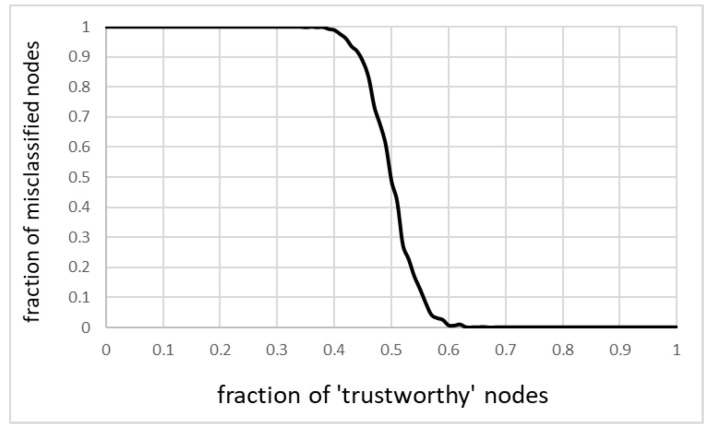
Misclassification as a function of the fraction of trustworthy nodes.

Figure 6 shows misclassification as a function of a fraction of untrustworthy nodes. The relationship is not linear, but there is a rapid transition from correct to incorrect classifications when the number of untrustworthy (faulty) nodes reaches and then exceeds half of all the nodes. The growth is increasingly fueled by untrustworthy nodes being classified as trustworthy ones (specifically that the number of trustworthy nodes decreases).

It is encouraging that the model was able to deliver correct classification despite reasonably high fraction of faulty nodes. It only confirms that it can be used to detect faulty nodes for as long as its number is not overwhelming.

### 6.4. Simulation Results: Response to a Rapid Transition

The simulation also covered situations where a given node transited from being trustworthy to being untrustworthy, or vice versa. Specifically, the situation where the fully trustworthy node, correctly reporting the value, suddenly becomes a faulty one, was of interest. The model was always able to respond to the transition, but the way it responded as well as the speed of the response varied.

**Figure 7:**
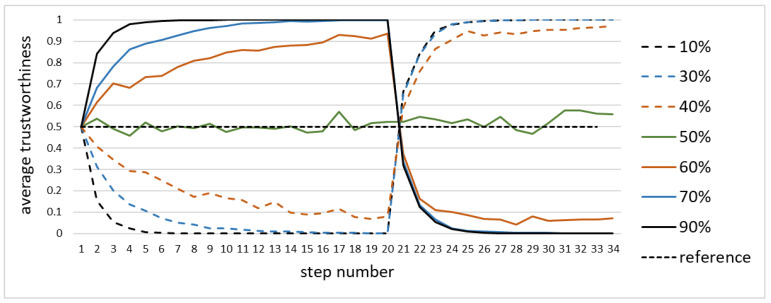
Response to transition as a function of the fraction of trustworthy nodes.

Figure 7 shows the simulation where the single node transited from reporting 1.0 to reporting 0.0 at the 20th step, as a function of the fraction of trustworthy nodes. The response was always fast, and it took approximately seven steps to correctly re-asses the trustworthiness of the node. Should the 0–1 classifier were used, then the re-classification would have required only two to three steps. The further is the fraction of untrustworthy nodes from 50%, the longer it took.

For the fractions of trustworthy nodes above 50% both the original assessment of trustworthiness and the new one after the transition are correct. Higher fractions of untrustworthy nodes made both of them inverse.

## 7. Discussion and Conclusions

This paper discusses the use of the reputation-based model (NUT: Network-Uncertainty-Trust) to decrease the uncertainty of measurement in dense, autonomous, heterogeneous IoT networks of sensor nodes that measure some physical phenomena. The main contribution of this paper lies in proposing, and then analyzing, an algorithm to calculate reputation of sensors that does not require external observers or reference measurements.

Social reputation affects social behavior as it tends to be used as an indicator that people take into account while choosing others to engage with. In the sensor network, sensors do not have a choice of engaging with or ignoring other sensors. Still, the reputation can be used as follows:

- The network as a whole is primarily concerned with the understanding of the overall situation, and for that, it synthesizes data from sensors into a general assessment of the phenomenon. The reputation can be used by this assessment so that data from sensors of lower reputation will contribute less to an overall outcome, thus increasing the overall certainty of its assessment.
- Even if the impact of data from untrustworthy nodes can be suppressed, their existence negatively affects the overall quality of the network. Therefore a synthetic metric (such as the unit response) taken off of all reputations will allow to determine whether the network as a whole performs well, or whether one of several networks is more trustworthy than others.
- Finally, reputation can help to determine weak spots that require attention. Even though the reputation itself cannot tell whether it is an unintended fault or the malicious attack, it allows the maintenance to concentrate on selected nodes so that any quality problems can be dealt with more rapidly.

It has already been mentioned that the method presented here may not be the best choice for all sensor networks. The authors can see its applicability for networks with relative over-provision of sensors, with a dense network of them, measuring natural phenomena. Its application to other types of networks has to be verified.

There is an inherent limitation in the use of the model in that it operates well only when the fraction of trustworthy nodes exceed half of the population. This may limit its use in situations where there are not enough trustworthy nodes in the network. The authors currently work on solutions that may provide certain relief to such situations. Not pre-empting detailed research, Figure 7 indicates that by improving the network design and maintenance it is possible to achieve significant improvements even for a small fraction of trustworthy nodes.

**Figure 8:**
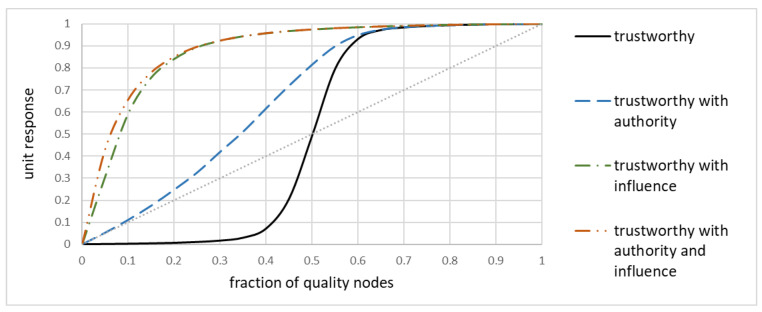
Unit response for various additional properties.

Figure 8, similarly to Figure 4a, shows the unit response as a function of a fraction of trustworthy nodes. The original sigmoid-like shape is marked as ‘trustworthy.” Two improvements have been made. First, all trustworthy nodes were made authority ones, i.e., their trustworthiness was never questioned by other nodes. Second, some of the trustworthy nodes became “influencers” by providing frequent opinions about several other nodes. The first improvement made the unit response always better than the no-weighted average, even for very small number of trustworthy nodes. However, it is the influence that really matters: if a trustworthy node (whether authority or not) can issue its opinions frequently and for several nodes, then the network as a whole benefits.

While this is a promising research direction, further studies must be made to understand the relationship between the structure and maintenance of the network and unit response, so that even networks with only few reference nodes could benefit from it.

Current work, apart from studying the impact of those improvements, focuses on implementing and verifying the model with data collected from the actual heterogeneous network. In addition, the question of graph structural properties attracted current research interest of the authors. Reputation works best if there are no cliques or clusters of opinions i.e., where opinions arrive from the whole network about the whole network. This may not be the case for the IoT networks where the problem of clustering happens in situations where a limited number of sensors, because of the nature of the physical phenomena, form a clique. In the absence of additional information, it is not always possible to resolve such situations.

This paper does not elaborate on the important issue of eventual convergence, i.e., the property of the model to eventually reach the stable assessment of trustworthiness of the node, provided that nodes will not change their actual trustworthiness. While the simulation indicates that it may be indeed the case (e.g., see Figure 7), this subject is covered in detail by a separate paper, in preparation [35].

There, convergence is analyzed in three stages. First, simulation runs are used to develop more detailed intuition about the expected behavior of the function for graphs with random opinions and nodes that are stable in their level of trustworthiness. Then, the simplification of the opinion graph allows for the analytical treatment, allowing for the formulation of the iterative function that expresses the sequence of assessments under stable conditions. The final stage is the mathematical proof, based on demonstrating that, for the applicable range of parameters, this function satisfies properties of Cauchy sequences, therefore it converges.
